# The Utility of Leadless Atrioventricular Synchronous Pacemaker Implantation as a Novel Alternative to a Traditional Pacemaker During Pregnancy

**DOI:** 10.19102/icrm.2023.14084

**Published:** 2023-08-15

**Authors:** Meet Shah, Ashkan Hashemi, Felix Afriyie, Inderjit Singh, Hyoeun Kim, Emad F. Aziz

**Affiliations:** ^1^Department of Cardiology, Rutgers New Jersey Medical School, Newark, NJ, USA

**Keywords:** Leadless pacemaker, Micra™, pregnancy

## Abstract

The Micra™ leadless pacemaker (Medtronic, Minneapolis, MN, USA) is indicated for the management of symptomatic bradycardia and advanced heart block. However, the safety of this procedure during pregnancy has not been studied. Here, we present a unique case of Micra™ leadless pacemaker implantation (MLP) in a 31 weeks pregnant patient with intermittent complete heart block who presented with multiple syncopal episodes. The patient underwent an MLP implantation with <40 mGy radiation exposure (about 0.6 min of total fluoroscopy time), which is deemed a negligible dose of radiation exposure during pregnancy. Her subsequent hospital course was uneventful, and she was safely discharged home and was able to continue her pregnancy and delivery without further syncopal episodes or incidence of heart block.

## Introduction

Symptomatic bradycardia and advanced heart block are cardiac electrical conduction diseases that require pacemaker implantation as a corrective measure. There are numerous predisposing factors that can make a patient more prone to having these arrhythmias. The patient in this case had a history of Chagas disease, which is considered a predisposing condition for developing conduction abnormalities. Several of the cardiac rhythm abnormalities more commonly seen in Chagas disease include right bundle branch block and first-, second-, and third-degree atrioventricular (AV) block.^1^ In addition, this patient had undergone an open-heart surgery to correct both mitral and aortic valve stenosis for rheumatic heart disease that could potentially cause heart block.^2^ The management of these conditions includes a pacemaker implantation. A well-known risk of a pacemaker implantation is exposure to radiation because of the use of fluoroscopy during the procedure. Hence, these procedures become a challenge to perform in pregnant patients.

According to the United States Nuclear Regulatory Commission (USNRC), the recommended maximum total fetus exposure during pregnancy should be <5.0 mSv.^3^ A fetal radiation dose of <50 mGy is considered safe, whereas a radiation dose of 50–100 mGy is considered inconclusive in terms of its impact on the fetus. Doses of >150 mGy are considered the minimum radiation dose capable of causing detrimental effects to a fetus, based on observation. Radiation exposure during various stages of a pregnancy affects the fetus in several ways, ranging from effects on organogenesis earlier in the pregnancy to miscarriage, growth reduction, intelligence quotient reduction, and severe neurological defects.^3^ Certainly, this presents a dilemma when treating a pregnant patient who requires a pacemaker for the management of heart block. Different approaches have been explored in the literature, such as performing 3-dimensional cardiac mapping to aid the implantation of a traditional dual-chamber pacemaker.^4^ During the coronavirus disease 2019 (COVID-19) pandemic, leadless pacemakers were shown to be a safer alternative to traditional pacemakers in light of their less-invasive nature, which allows for a shorter procedure time and a reduced mean fluoroscopy time.^5^ Here, we report an alternate novel approach to manage a pregnant patient with complete heart block by implanting a leadless pacemaker with minimal fluoroscopy exposure.

## Case presentation

**Figure 1:**
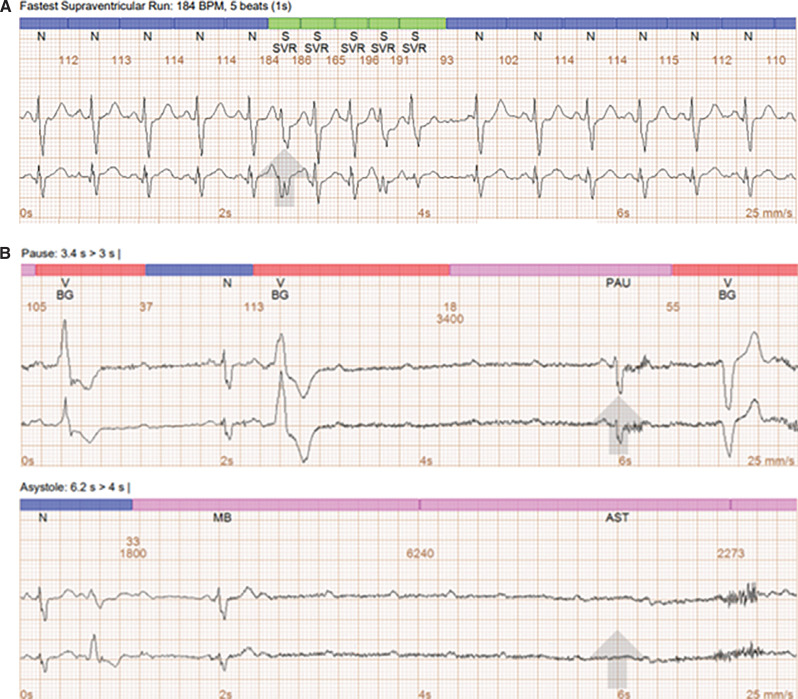
**A:** Event monitor rhythms illustrating the fastest supraventricular run at 184 bpm. **B:** Event monitor rhythms illustrating complete heart block.

A 39-year-old, 31 weeks pregnant female patient with a past medical history significant for Chagas disease and rheumatic heart disease with severe valvular involvement who underwent mitral valve replacement and aortic valve replacement and who presented with intermittent palpitations and syncope was referred to cardiology for evaluation. Her diagnostic work-up included a transthoracic echocardiogram that showed a normal ejection fraction and normal mechanical valve function. Two-week event monitoring showed numerous sinus bradycardia events: 66 episodes of missed heartbeats, 7 episodes of sinus pauses for >2 s each, and 3 episodes of complete heart block of 6–10 s long. The recurrent dizziness and syncopal episodes correlated with sinus bradycardia, and complete heart block rhythms were seen on the event monitor. Also, there were 26 episodes of tachycardia with an average heart rate of 123–184 bpm **(Figure 1A)**. The event monitor review showed intermittent symptomatic complete heart block. As she was pregnant, she was deemed a suitable candidate for Micra™ AV leadless pacemaker (Medtronic, Minneapolis, MN, USA) implantation as it would minimize exposure of both her and the fetus to radiation during fluoroscopy. For the pacemaker implantation, a right femoral vein approach was used to introduce the Micra™ leadless delivery system. The Micra™ AV leadless pacemaker was implanted into the right ventricle mid-septum, and electrical testing was performed to ensure AV tracking with narrow QRS and to ensure the functionality of the device. The total fluoroscopy time of the procedure was 0.6 min, with the patient exposed to 40 mGy of radiation. She was monitored for >24 h by the cardiology and the obstetrics and gynecology services. A postprocedure non-stress test was performed to evaluate the status of the fetus, which was reassuring. The patient was followed up with in the outpatient clinic 3 weeks after discharge, and she reported no significant symptoms. Her pacing burden was noted to be 0.7% with great AV tracking. She was able to continue her pregnancy and delivery without further syncopal episodes or incidence of heart block.

## Discussion

**Figure 2:**
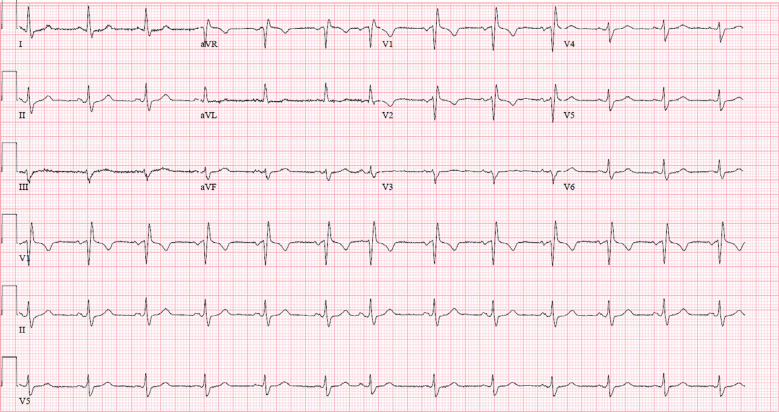
Electrocardiogram illustrating sinus rhythm at a rate of 76 bpm with incomplete right bundle branch block.

Several conduction abnormalities of the heart require invasive management with a pacemaker insertion. Patients with Chagas disease can present with many conduction abnormalities, including sick sinus syndrome, atrial extrasystoles, intra-atrial conduction disturbances, atrial fibrillation, or flutter in various stages.^6^ At the ventricular level, both conduction disturbances and arrhythmias are conspicuous expressions of myocardial damage. A right bundle branch block alone or in combination with left anterior hemiblock is the most common conduction defect seen in these patients **(Figure 2)**. These arrhythmias are usually aggravated by increased adrenergic and autonomic activity seen in pregnancy and can potentially become significant enough to limit uterine flow.^7^ Our patient, in addition to having a history of Chagas disease, had also undergone open-heart surgery for mechanical replacements of her mitral and aortic valves, which could have added to the fibrosis at the site of the AV node and could contribute to the heart block.

**Figure 3:**
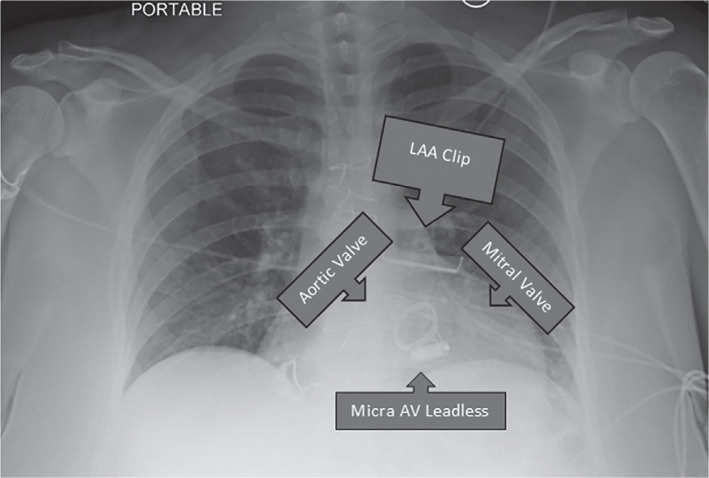
Chest X-ray film illustrating the leadless pacemaker device in the right ventricular cavity. *Abbreviations:* AV, atrioventricular; LAA, left atrial appendage.

Management of such conduction abnormalities during pregnancy is often challenging and requires a thoughtful risk–benefit analysis. In general, pregnant women have an increased risk of arrhythmias compared to other women of childbearing age.^8^ In most cases, invasive approaches are deferred to term pregnancy to avoid hazardous radiation exposure to the fetus, as they often do not result in significant hemodynamic changes.^9^ On the other hand, other case series suggest the potential progression of AV conduction block during pregnancy.^10^ Further, deferring intervention was not a valid choice for our patient who presented with recurrent episodes of syncope. An event monitor study revealed a long episode of AV conduction block up to 10 s, potentially hazardous to both mother and the fetus, necessitating a concrete treatment approach with a pacemaker. A leadless intracardiac transcatheter pacing system has been established as a safe alternative when compared to the traditional transvenous pacemaker, with fewer associated hospitalizations and system revisions.^11^ The Micra™ Atrial Tracking using a Ventricular Accelerometer (MARVEL) study showed that accelerometer-based atrial sensing is feasible and significantly improves AV synchrony in patients with AV block and a single-chamber leadless pacemaker implanted in the right ventricle with AV tracking up to 88%.^12^ We decided to choose a leadless pacemaker over a traditional transvenous pacemaker to minimize the radiation exposure to the fetus. The mid-septum in the RV was chosen as the implantation site, as shown in **Figure 3**, as some data suggest this to be an optimal site associated with narrowest paced QRS.^13^ Additionally, our young patient was mostly expected to require pacing for her symptomatic arrhythmia for the remainder of the pregnancy only. If her pacing burden was significantly higher at follow-up visits postpregnancy, a clinical decision would be made to change it to a traditional pacemaker eventually. The physician undertaking the procedure had extensive experience with leadless pacemakers and an overall extreme level of comfort with the procedure, having performed >300 such device implantations. The leadless device was successfully implanted with approximately 0.6 min of fluoroscopy time and total radiation exposure of <40 mGy, which is considered a negligible amount of radiation exposure for pregnant patients per the USNRC recommendations, without any complications.

Leadless pacemaker implantation could be an alternative approach to traditional pacemaker implantation in pregnant patients, mainly due to the minimally invasive approach, lack of stimulation leads, reduced risk of site infection, and (as particularly seen in this case) limited radiation exposure time.^14,15^ To the best of our knowledge, this is the first reported case of leadless AV pacemaker placement in a pregnant patient. The safety profile of the leadless pacemaker procedure, as seen in this case, suggests that it can be a favorable treatment option for pregnant patients with conduction abnormalities requiring a cardiac pacemaker.

## Conclusion

Leadless AV pacemaker placement is a safe treatment option for pregnant patients with conduction abnormalities requiring permanent pacing when performed at a highly resourced center for advanced care.

## References

[1] Ribeiro AL, Nunes MP, Teixeira MM, Rocha MO (2012). Diagnosis and management of Chagas disease and cardiomyopathy. Nat Rev Cardiol.

[2] Goel PK, Moorthy N, Bhatia T (2013). Rheumatic severe mitral stenosis with complete heart block. Pediatr Cardiol.

[3] Yoon I, Slesinger TL (2023). Radiation exposure in pregnancy. StatPearls [Internet].

[4] Elbadawi A, Arif Z, Chuprun D, Rao M (2016). Permanent pacemaker implantation without fluoroscopy in a pregnant woman with complete atrioventricular block: a case report. Indian Pacing Electrophysiol J.

[5] Kazmi M, Rashid S, Markovic N, Kim H, Aziz EF (2021). Micra™ leadless intracardiac pacemaker implantation: a safer option during the coronavirus disease 2019 pandemic. J Innov Card Rhythm Manag.

[6] Rojas LZ, Glisic M, Pletsch-Borba L (2018). Electrocardiographic abnormalities in Chagas disease in the general population: a systematic review and meta-analysis. PLoS Negl Trop Dis.

[7] Hayes C (2013). A review of arrhythmias in pregnancy. BHRS Editorials.

[8] McAnulty JH (2012). Arrhythmias in pregnancy. Cardiol Clin.

[9] Keepanasseril A, Maurya DK, Suriya Y, Selvaraj R (2015). Complete atrioventricular block in pregnancy: report of seven pregnancies in a patient without pacemaker. BMJ Case Rep.

[10] Thaman R, Curtis S, Faganello G (2011). Cardiac outcome of pregnancy in women with a pacemaker and women with untreated atrioventricular conduction block. Europace.

[11] Reynolds D, Duray GZ, Omar R (2016). A leadless intracardiac transcatheter pacing system. N Engl J Med.

[12] Steinwender C, Khelae SK, Garweg C (2020). Atrioventricular synchronous pacing using a leadless ventricular pacemaker: results from the MARVEL 2 study. JACC Clin Electrophysiol.

[13] Sharma P, Singh Guleria V, Bharadwaj P, Datta R (2020). Assessing safety of leadless pacemaker (MICRA) at various implantation sites and its impact on paced QRS in Indian population. Indian Heart J.

[14] Lancellotti P, Gach O, Marechal P, Robinet S (2019). Pacemaker miniature sans sonde de type Micra^®^ [Micra^®^ leadless pacemaker]. Rev Med Liege.

[15] El-Chami MF, Al-Samadi F, Clementy N (2018). Updated performance of the Micra transcatheter pacemaker in the real-world setting: a comparison to the investigational study and a transvenous historical control. Heart Rhythm.

